# Papillitis in Neurosyphilis

**Published:** 2019

**Authors:** Mioara-Laura Macovei, Raluca-Diana Georgescu

**Affiliations:** * Ophthalmology Department, “Dr. Carol Davila” Central Military Emergency University Hospital, Bucharest, Romania

**Keywords:** papillitis, optic nerve pathology, visual field defects, anterior uveitis

## Abstract

We present a case of a 47-year-old female patient, with papillitis in the right eye and anterior uveitis in both eyes, as a manifestation of untreated neurosyphilis.

## Introduction

Syphilis is an infectious disease caused by Trepona pallidum [**1**]. This pathology was called “the great imitator” because it may cause symptoms similar to other diseases [**2**]. It has 3 stages: primary, secondary, tertiary. Each stage has its own clinical signs and symptoms: primary syphilis - chancre, secondary syphilis - macular papular rash, lymphadenopathy, mucosal ulceration, tertiary syphilis - gummas, cardiac and neurological symptoms. There is also latent syphilis with no clinical manifestations but detectable by serological tests. The bacterium can affect the central nervous system and result in neurosyphilis, which can occur at any stage of the disease. If the disease is left untreated, it has a mortality rate of 8% to 58% [**1**,**2**].

## Case report

We present the case of a 47-year-old female patient, who came in our hospital complaining of sudden and severe decrease in visual acuity in the right eye for two weeks, accompanied by headache and moderate continuous pain in the right eye. For the medical history, we could mention medically controlled hypertension and a neglected hyperthyroidism. In addition, the patient had a history of penicillin allergy.

At presentation, her best-corrected visual acuity was RE counting fingers (CFs), LE: 1. The IOP was normal in both eyes, BE 16 mmHg on non-contact tonometry.

**Fig. 1:**
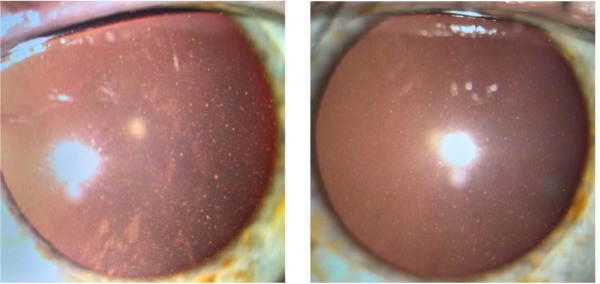
Multiple, small endothelial precipitates in both eyes

**Fig. 2:**
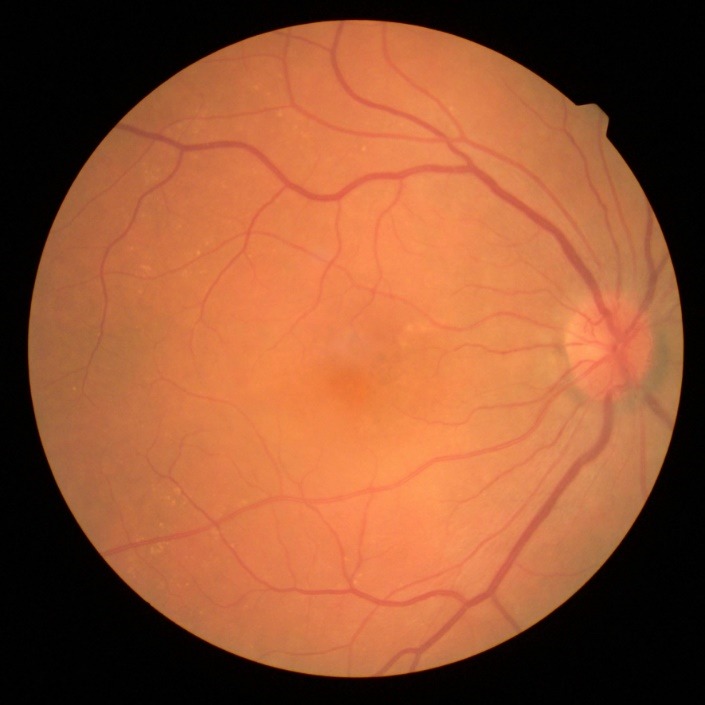
Hyperemic optic disc with blurred margins and macular pigmentary abnormalities in the right eye

**Fig. 3:**
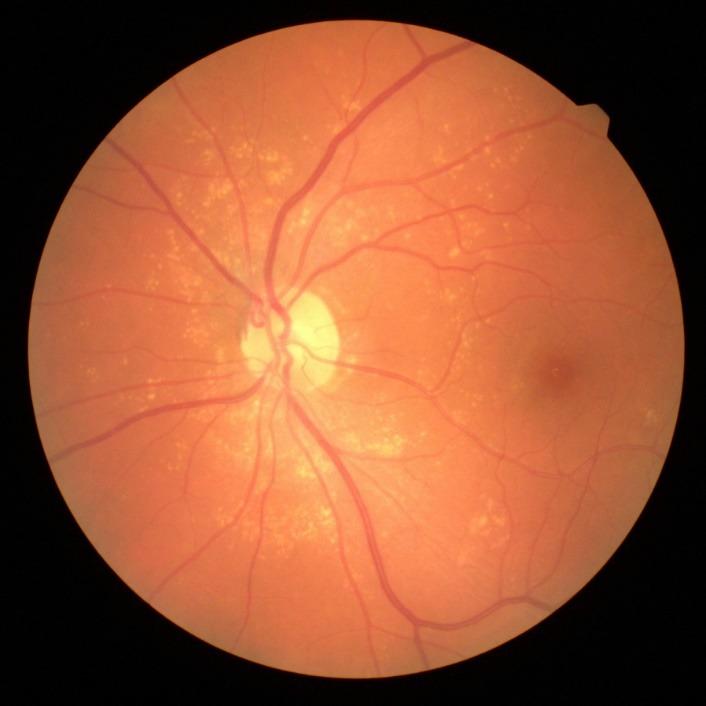
Drusen along the vascular arcades and in the macular region in the left eye

Slit-lamp examination of the anterior segment revealed multiple small endothelial precipitates in both eyes (**Fig. 1**) and a Relative Afferent Pupillary Defect (RAPD) present in the right eye. The examination of the posterior pole showed hyperemic optic disc with blurred margins and macular pigmentary abnormalities in the right eye (**Fig. 2**), drusen along the vascular arcades and in the macular region in the left eye (**Fig. 3**).

Ishihara test showed abnormal color vision in the right eye.

**Fig. 4:**
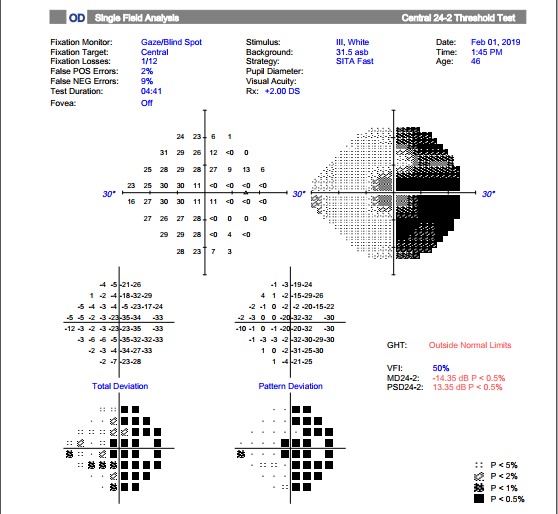
Temporal hemianopsia in the right eye

**Fig. 5:**
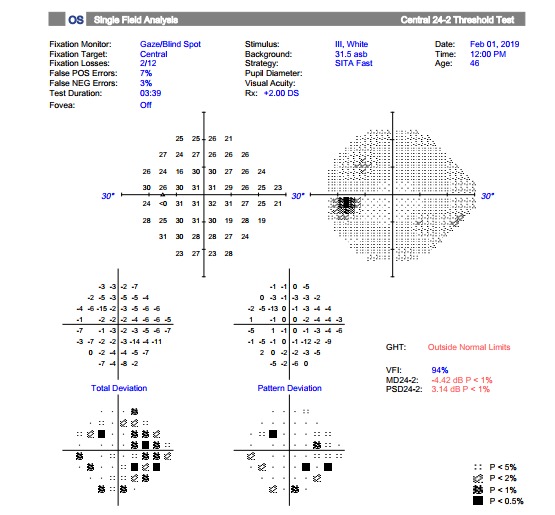
Multiple non-systematized defects in all the four quadrants in the left eye

The grayscale and pattern deviation plots from a Humphrey 24-2 Central Threshold Test using SITA-Standard software showed temporal hemianopsia in the right eye (**Fig. 4**) and multiple non-systematized defects in all the four quadrants in the left eye (**Fig. 5**).

**Fig. 6:**
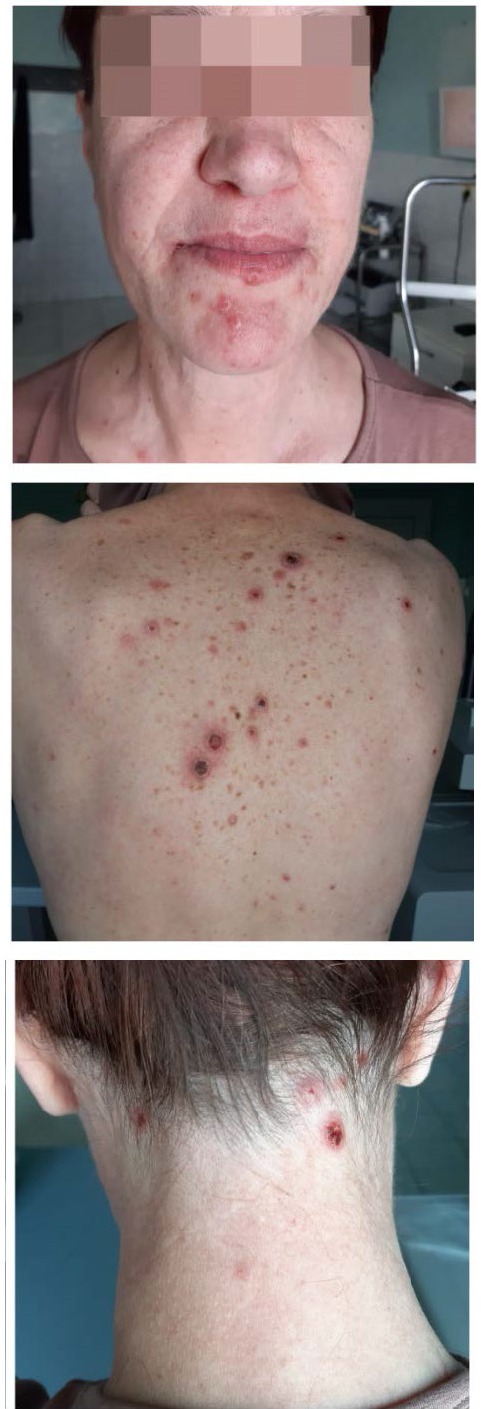
Plaques > 10 mm with hemorrhagic crusts

On clinical examination, the patient had plaques > 10 mm with hemorrhagic crusts on the body, neck, and head (**Fig. 6**).

Further blood tests were ordered: complete count blood (CBC), a metabolic panel and a lipid panel, rheumatoid factor, ANA, ANCA, and serum ACE, HLA B27, IgM and IgG for Toxoplasma gondii. The next analyses were raised: fibrinogen - 617.0 mg/ dl (276.00-417.00), erythrocyte sedimentation rate (ESR) - 40 mm/ 1 h (1.00-25.00), C reactive protein - 28.68 mg/ l (0.00-5.00) and blood glucose - 303 mg/ dl (74.00-106.00). Her viral serology was negative for HIV, hepatitis, herpes simplex. TPHA and VDRL were positive.

The otorhinolaryngology and neurological exam were normal and the head and orbit MRI with i.v. contrast was also normal. The diagnoses of type II diabetes mellitus and hyperthyroidism was established by the endocrinological exam and the patient received adequate treatment.

The dermatologic consult suspected lymphomatoid papulosis and tertiary syphilis, but the skin biopsy necessary for the diagnosis of lymphomatoid papulosis was postponed because of the positive test results for Syphilis. 

A lumbar puncture was performed with a normal opening pressure. Cerebrospinal fluid protein and glucose were both raised and TPHA and VDRL were positive. The patient was diagnosed with neurosyphilis.

The patient received alternative treatment with 200 mg doxycycline p.o and 2 g ceftriaxone i.v 14 days, because of her penicillin allergy.

After 7 days of treatment her best-corrected visual acuity was RE: 0,4 nc, LE: 1. The visual field examination in the right eye showed that the defect from presentation decreased and the defects in the left eye disappeared.

**Fig. 7:**
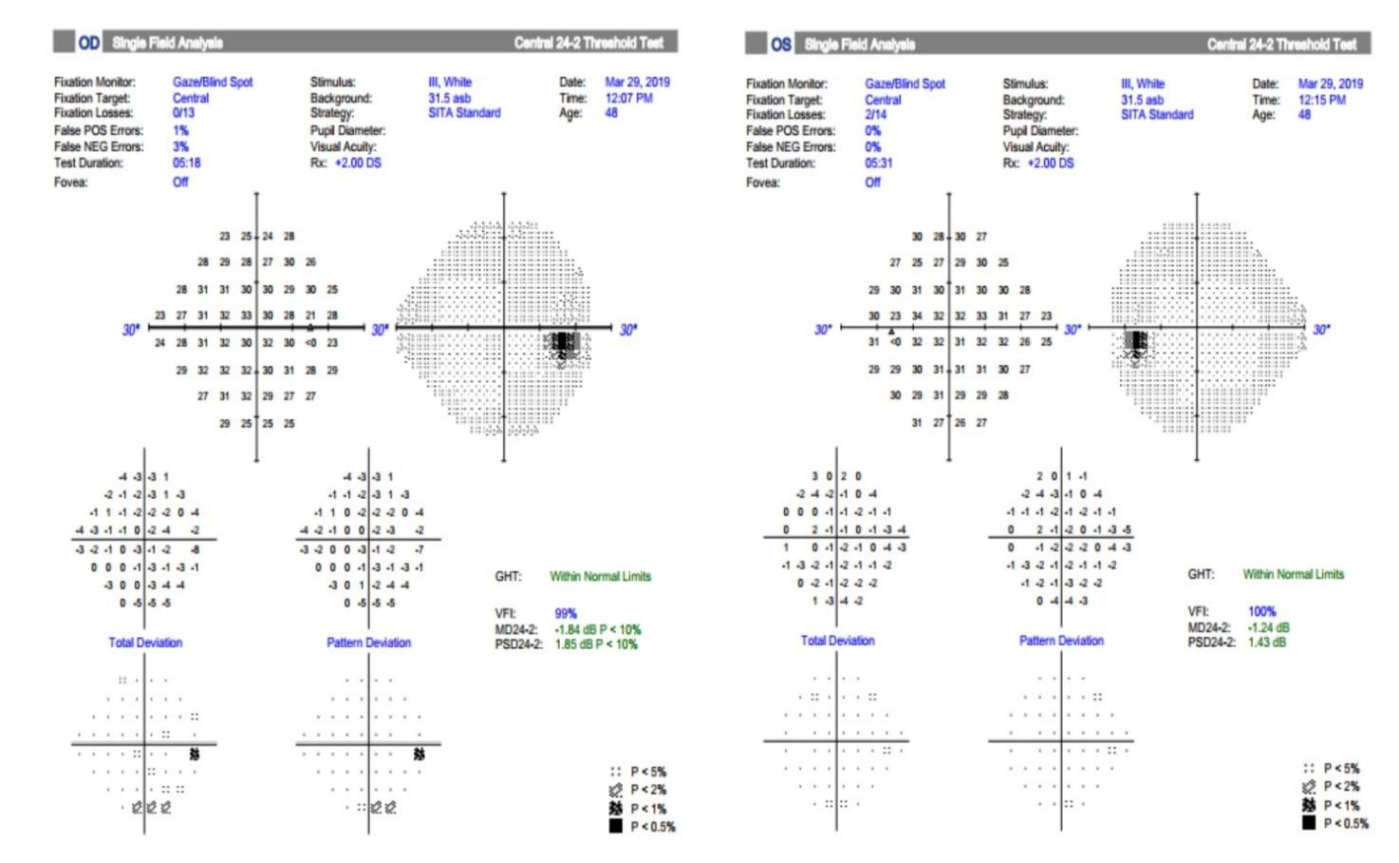
Visual fields in both eyes were within normal limits after one month

**Fig. 8:**
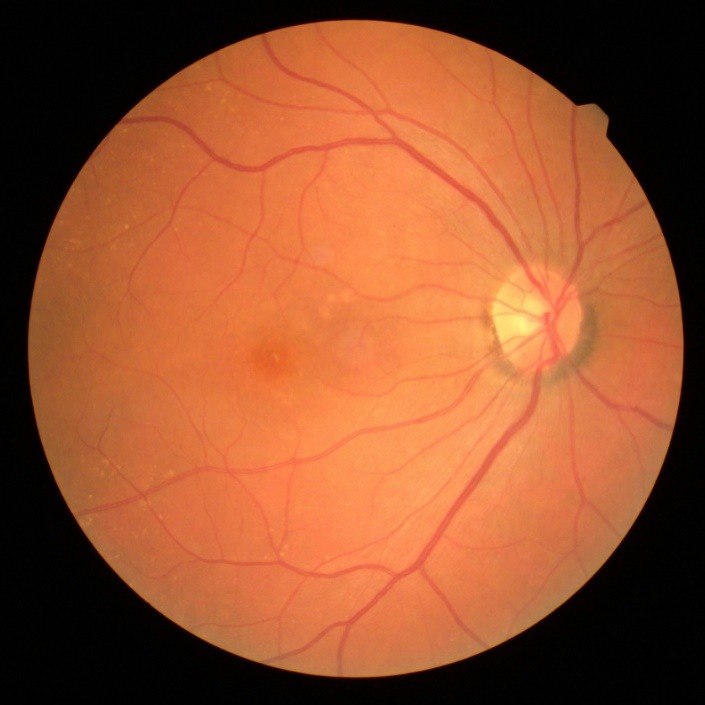
Normal aspect of the optic disc after treatment

The next follow-up was after one month and her BCVA was BE: 1 and the visual fields in both eyes were within normal limits (**Fig. 7**). The endothelial precipitates disappeared and the aspect of the optic disc in the right eye was normal (**Fig. 8**).

## Discussion

Papillitis or optic neuritis is the inflammation and deterioration of the anterior portion of the optic nerve known as the optic disc [**3**-**5**]. The diffuse margins of the optic disc suggested papillitis, papilledema, or anterior ischemic optic neuropathy: papilledema was excluded due to normal opening pressure of the CSF. Some exclusion factors for arteritic AION were age < 70 years old, absence of jaw claudication, absence of the pale (chalky) aspect of the optic disc and for nonarteritic AION were age < 60 years old, absence of a crowded disc, and the pain that accompanied the visual loss [**7**]. 

Papillitis has many causes including multiple sclerosis, viral or bacterial infections, nutritional or metabolic disorders such as diabetes mellitus and hyperthyroidism [**6**]. The diagnostic of syphilitic papillitis was performed based on serological positive test, clinical ocular manifestations and the examination and culture of the cerebrospinal fluid. In medical literature, only a few cases of syphilitic papillitis were described in immunocompetent patients.

Anterior uveitis is a more common ocular manifestation than papillitis in syphilis. Anterior uveitis occurs in about 4% of the patients with secondary syphilis; it may be granulomatous or non-granulomatous and is bilateral in 50% of the cases [**6**].

The alternative treatment in patients with neurosyphilis and allergy to penicillin is with ceftriaxone 2 g i.v. for 10-14 days and with doxycycline 200 mg p.o twice daily for 28 days [**8**,**9**].
